# Vestibular rehabilitation therapy on balance and gait in patients after stroke: a systematic review and meta-analysis

**DOI:** 10.1186/s12916-023-03029-9

**Published:** 2023-08-25

**Authors:** Lijiao Meng, Qiu Liang, Jianrong Yuan, Siyi Li, Yanlei Ge, Jingyi Yang, Raymond C C Tsang, Quan Wei

**Affiliations:** 1 Department of Rehabilitation Medicine and Institute of Rehabilitation Medicine, West China Hospital, Sichuan University, No. 37, Guo Xue Alley, Chengdu, Sichuan 610041 China; 2 Key Laboratory of Rehabilitation Medicine in Sichuan Province, West China Hospital, Sichuan University, No. 37, Guo Xue Alley, Chengdu, Sichuan 610041 China; 3 Department of Rehabilitation Medicine, Chongqing University Three Gorges Hospital, No. 165. Xin Cheng Road, Chongqing, China; 4 Department of Rehabilitation Sciences, The Hong Kong Polytechnic University, No. 11 Yuk Choi Road, Hung Hom, Kowloon, Hong Kong, China

**Keywords:** Vestibular rehabilitation, Stroke, Balance, Gait, Systematic review, Meta-analysis

## Abstract

**Background:**

There is limited evidence to support the use of vestibular rehabilitation therapy (VRT) on improving balance and gait in patients after stroke. This systematic review aimed to evaluate the effects of VRT in addition to usual rehabilitation compared with usual rehabilitation on improving balance and gait for patients after stroke.

**Methods:**

This review followed the Preferred Reporting Items for Systematic reviews and Meta-Analysis statement guidelines. Ten electronic databases were searched up to 1 June 2023 without restrictions in language and publication status. The PEDro scale and the Grading of Recommendations Assessment Development, and Evaluation were used to evaluate the risk of bias and the certainty of evidence. The meta-analysis was conducted with Review Manager 5.3.

**Results:**

Fifteen randomised controlled trials with 769 participants were included. PEDro scale was used to assess the risk of bias with a mean score of 5.9 (0.7). VRT was effective in improving balance for patients after stroke (SMD = 0.59, 95% CI (0.40, 0.78), *p* < 0.00001), particularly for patients after stroke that occurred within 6 months (SMD = 0.56, 95% CI (0.33, 0.79), *p* < 0.00001) with moderate certainty of evidence. Subgroup analysis showed that VRT provided as gaze stability exercises combined with swivel chair training (SMD = 0.85, 95% CI (0.48, 1.22), *p* < 0.00001) and head movements (SMD = 0.75, 95% CI (0.43, 1.07), *p* < 0.00001) could significantly improve balance. Four-week VRT had better effect on balance improvement (SMD = 0.64, 95% CI (0.40, 0.89), *p* < 0.00001) than the less than 4-week VRT. The pooled mean difference of values of Timed Up-and-Go test showed that VRT could significantly improve gait function for patients after stroke (MD =  −4.32, 95% CI (−6.65, −1.99), *p* = 0.0003), particularly for patients after stroke that occurred within 6 months (MD =  −3.92, 95% CI (−6.83, −1.00), *p* = 0.008) with moderate certainty of evidence.

**Conclusions:**

There is moderate certainty of evidence supporting the positive effect of VRT in improving balance and gait of patients after stroke.

**Trial registration:**

PROSPERO CRD42023434304

**Supplementary Information:**

The online version contains supplementary material available at 10.1186/s12916-023-03029-9.

## Background

Patients with stroke are at high risk of falling due to impairments in motor and higher cerebral functions [1]. The vestibular dysfunction, sensory impairment or perceptual dysfunction after stroke may lead to an increased risk of falling [2, 3]. Therefore, neurorehabilitation aimed at improving postural stability and balance has received considerable attention in clinical practice.

Vestibular rehabilitation therapy (VRT) is an exercise-based therapy that aims to promote gaze stability, improve postural stability and facilitate sensory integration for patients [4, 5]. VRT has been found to be effective in improving balance in patients with peripheral vestibular dysfunction [6, 7] and individuals with vestibular hypofunction [8–10]. VRT also appears to be an effective intervention in enhancing balance and postural recovery in individuals after damage of the central nervous system [11], including Parkinson’s disease [12], multiple sclerosis [13, 14], concussion [15] and cerebral palsy [16]. The effects of vestibular rehabilitation on gait performance in patients after stroke had been evaluated in a systematic review without meta-analysis [17]. However, only three studies were included in qualitative synthesis, and the overall certainty of evidence assessed by the Grading of Recommendations Assessment, Development and Evaluation (GRADE) criteria was very low. Definitive conclusions on the effectiveness of VRT on gait performance in patients after stroke could not be made. Therefore, the present systematic review aimed to evaluate the effects of VRT in addition to usual rehabilitation (UR) compared with UR on improving balance and gait for patients after stroke by searching for evidence from new randomised controlled trials (RCTs) with data synthesis.

## Methods

The systematic review was conducted according to the Preferred Reporting Items for Systematic reviews and Meta-Analysis statement (Additional file 1) [18]. This review was registered in PROSPERO (ID: CRD42023434304).

### Search strategy

Electronic databases, including PubMed, EMBASE, Web of Science, Cochrane Central Register of Controlled Trials, Physiotherapy Evidence Database (PEDro), Cumulative Index to Nursing and Allied Health Literature, China Biology Medicine database, China National Knowledge Internet, VIP database and Wanfang database were searched to identify published RCTs of VRT for patients after stroke. The date of the search was from the earliest available to 1 June 2023, without restrictions in language and publication status. The references of identified articles were searched to ensure comprehensiveness. The search terms combined Medical Subject Headings and the keywords (vestibular, stroke, balance, posture, walking and gait). Chinese synonyms were searched in Chinese databases. The search strategy used in PubMed is presented in Additional file 2.

### Eligibility criteria

#### Participants

RCTs were included if the participants (1) were diagnosed with stroke and (2) verified impaired balance and gait due to stroke. Studies were excluded if the participants (1) presented neurological or orthopaedic problems unrelated to stroke that would affect postural stability, (2) balance or gait impairments prior to the stroke and (3) visual field defects.

#### Intervention

Studies evaluating VRT in addition to UR compared with UR were eligible for inclusion. VRT includes at least one of the following vestibular training strategies: gaze stability exercises (GSE), eye-head movements, head movements, vestibular stimulation, specific exercises or techniques enhancing the vestibular function. UR refers to stroke rehabilitation programmes customised according to the identified problems of patients after stroke but did not include VRT.

#### Outcome measures

The following outcomes that assessed balance or gait were identified to examine the efficacy of VRT:

1. Balance measured by the Berg balance scale (BBS), Fugl–Meyer balance scale (FM-B), activities-specific balance confidence Scale (ABC), Brunel balance assessment (BBA) and postural assessment scale for stroke patients. Quantitative outcome measured by specific balance equipment were included as well. Fall events were used as a proxy indicator of balance.
2. Gait measured by the timed up-and-go test (TUG), 10-m walking test, dynamic gait index, functional gait assessment and gait parameters.

### Study selection and data extraction

The articles were independently searched and selected according to the eligibility criteria by two reviewers (QL and YG). Duplicate articles were removed. Titles and abstracts of searched studies were screened, and then studies with full texts were obtained to determine final inclusion. Another two reviewers (LM and JY1) extracted the data independently by using predesigned sheets created by Microsoft Excel. Data regarding the study demographics were extracted, including first author, publication year, information of participants (numbers, age and sex), and stroke characteristics (type, location of lesion, severity and duration of onset). Data on the intervention details and outcome measures were also extracted. Any disagreements about the study selection and data extraction were discussed until a consensus was reached or settled with the involvement of a third reviewer (JY2).

### Risk of bias and certainty of evidence evaluation

The PEDro Scale of the Physiotherapy Evidence Database [19] was used to assess the risk of bias of included studies. If there were no available scores of included studies in the PEDro database, two experienced reviewers (LM and QL) independently rated the study. The PEDro scale is a valid measure of the methodologic quality of clinical trials [20]. There are 11 items in the scale, with a total score ranging from 0 to a maximum of 10. Studies were considered high quality with a PEDro score of 6 or above and of moderate quality when the score was 4 or 5 [21]. The GRADE approach was used to evaluate the certainty of evidence by two experienced reviewers (LM and QL) independently. The GRADE system is currently the most widely used tool for grading the certainty of evidence in systematic reviews and clinical practice guidelines. The GRADE system specifies four categories for the certainty of a body of evidence as ‘very low’, ‘low’, ‘moderate’ or ‘high’ based on certain criteria [22]. Factors downgrading the certainty of evidence (risk of bias, inconsistency, indirectness, imprecision and publication bias) or those upgrading the certainty (large effect, plausible confounding and dose-response) were evaluated [23]. When there was any disagreement on the ratings between the two reviewers, a third reviewer (RCCT) was consulted.

### Data synthesis and analysis

The statistical analysis was performed using Review Manager (RevMan), version 5.3. Data synthesis was conducted when at least two comparable trials were available. The means and standard deviations (SDs) of the change between baseline and post-intervention were used to estimate the pooled effect. We estimated the means and SDs of change using the method described in the Cochrane Handbook Version 6.3 [24] in case only baseline and post-intervention values were available. The unreported means and SDs were calculated from the median, sample size and range and/or IQR using the methods introduced by Wan et al. [25]. We contacted authors via email to obtain the unreported data. The mean differences (MDs) or standardised mean differences (SMDs) with 95% confidence intervals (CIs) were calculated for continuous variables. The risk ratio was calculated for dichotomous variable. *P* values less than 0.05 were considered statistically significant, and 95% CIs were reported.

Statistical heterogeneity among studies was evaluated by the *χ*^2^ test (*p* = 0.10) and quantified by the *I*^2^ statistic test. An *I*^2^ is considered as “might not be important” for value of < 40%; as ‘may represent moderate heterogeneity’ for value of 30% to 60%; as ‘may represent substantial heterogeneity’ for value of 50 to 90%; and as ‘considerable heterogeneity’ for value of 75 to 100% [26]. Meta-analysis was performed using a random effects model, as there could be between-study variability due to variations in the stroke characteristics or applications of the VRT interventions. If heterogeneity was considered as moderate to substantial, subgroup analysis was applied.

Clinical heterogeneity among studies was assessed based on stroke characteristics, intervention characteristics and outcome measures. The effects of VRT on balance and gait domains were analysed respectively. In the data synthesis of balance and gait, studies were stratified into the duration of onset of stroke ‘within 6 months’ and ‘beyond 6 months’, according to the data available in those included studies and improvement potential of stroke. Given the diverse VRT protocols among studies, clinical heterogeneity was likely and subgroup analyses were performed for different types and intervention duration of VRT. When there were multiple outcome measures used for measuring the outcome domain in a study, the performance-based outcome measures were preferred and selected for analysis. For example, the BBA was chosen rather than the ABC in the study of Wang YQ [27].

## Results

### Flow of studies through the review

**Fig. 1:**
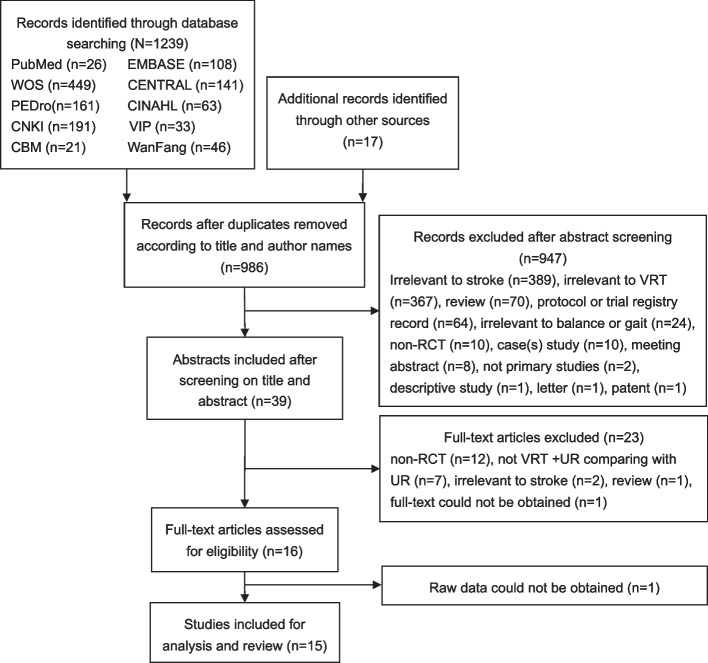
Preferred Reporting Items for Systematic Reviews and Meta-Analyses flow diagram for selection of studies. WOS Web of Science, CENTRAL Cochrane Central Register of Controlled Trials, PEDro Physiotherapy Evidence Database, CINAHL Cumulative Index to Nursing and Allied Health Literature, CNKI China National Knowledge Internet, VIP VIP database, CBM China Biology Medicine database, WanFang Wanfang database, RCT randomized controlled trial, VRT vestibular rehabilitation therapy, UR usual rehabilitation

A total of 1256 citations, of which 1239 citations from 10 databases and 17 citations form other sources, were identified to be potentially relevant for this review. After screening the titles and abstracts with removal of the duplicates, 39 full-text studies were retrieved and assessed. A total of 15 RCTs [27–41] with 769 patients met the inclusion criteria and were included in the meta-analysis (Fig. 1).

### Characteristics of studies

There were 6 (40.0%) articles [28–32, 39] written in English and 9 (60.0%) articles [27, 33–38, 40, 41] in Chinese (two [27, 40] of studies were theses). There were 6 (40.0%) articles [28, 30, 35, 39–41] investigated the effect of VRT on both balance and gait, 7 (46.7%) [27, 29, 33, 34, 36–38] on balance and 2 (13.3%) [31, 32] on gait.

Table 1 describes the characteristics of the included trials. There were 2 (13.3%) RCTs [30, 31] included patients with stroke duration beyond 6 months. There were 10 (66.7%) RCTs [27, 29, 32, 34, 36–41] included patients with the onset of stroke within 6 months and 4 (26.7%) [27, 37, 38, 40] of them included patients with stroke duration within 3 months. There was 1 (6.67%) RCT [28] with stroke that occurred within 3 to 15 months, and 2 (13.3%) RCTs [33, 35] did not state the duration of onset of stroke. These 3 RCTs could not be categorised according to the criterion of duration of onset of stroke within or beyond 6 months. The most commonly used outcome measurements were the BBS [28, 30, 35–41] (60.0%) and TUG [28, 32, 39, 40] (26.7%).

**Table 1:** Study characteristic

**Study (author, year)**	**Participants**			**Stroke characteristics**				**Outcome measures related to balance and gait**
	**Size (*n*)**	**Age (years)**	**Gender (M/F)**	**Duration (days)**	**Type (H/I)**	**Lesion side (L/R)**	**Lesion location**	
Correia, 2021 [28]	E: 33	73.3 ± 6.5	23/10	198.0 ± 77.9	5/28	/	/	Number of falls, BBS and TUG
	C: 35	73.5 ± 6.2	23/12	178.0 ± 66.6	7/28	/	/	
Dai, 2013 [29]	E: 24	57.2 ± 12.2	16/8	56.9 ± 38.9	/	/	/	Number of falls and PASS
	C: 24	64.5 ± 14.7	12/12	73.9 ± 37.9	/	/	/	
Elhamrawy, 2021 [31]	E: 16	66.5 ± 3.2	11/5	264.3 ± 46.5	4/12	/	/	Walking speed, walking cadence, SL-AS, SL-US and step width identified by using the Microsoft Kinect V2
	C: 16	68.5 ± 3.8	10/6	260.1 ± 40.8	5/11	/	/	
Guo, 2022 [40]	E: 14	61.07 ± 9.09	8/6	14.2 ± 4.7	6/8	/	/	BBS, TUG, 10MWT (seconds), APDCOP
	C: 14	62.64 ± 9.19	7/7	15.1 ± 4.7	5/9	/	/	
Hansson, 2020 [30]	E: 19	71.0 ± 11.1	8/11	255.0 ± 327.0	/	11/16 (no paresis: 5)	/	ABC, BBS and FGA
	C: 13	69.0 ± 10.4	5/8		/		/	
Huang, 2019 [33]	E: 20	54.6 ± 8.4	26/14	/	16/24	/	/	FM-B
	C:20			/		/	/	
Jiang, 2012 [34]	E: 48	50.7 ± 9.2	59/37	82.5 ± 26.1	/	/	/	FM-B
	C: 48				/	/	/	
Li, 2022 [35]	E: 42	57.92 ± 2.01	25/17	/	13/29	27/15	/	BBS, gait parameters (SL-AS, ST-AS and SW-AS) assessed by the Gait Watch
	C: 42	57.34 ± 2.11	24/18	/	11/31	26/16	/	
Mitsutake, 2017 [32]	E: 14	67.6 ± 9.0	11/3	52.4 ± 26.4	/	6/8	Supratentorial/infratentorial: 10/4	10MWT (m/s), TUG and DGI
	C: 14	68.1 ± 13.5	11/3	64.1 ± 37.7	/	7/7	Supratentorial/infratentorial: 10/4	
Wang YM, 2022 [36]	E: 17	66.0 ± 5.4	15/2	76.0 ± 33.9	4/13	5/12	/	BBS
	C:17	67.8 ± 4.1	14/3	90.8 ± 37.1	2/15	8/9	/	
Wang YQ, 2022 [27]	E: 28	51.93 ± 8.14	14/14	34.3 ± 21.5	15/13	15/13	/	BBA, ABC
	C: 27	50.52 ± 9.90	15/12	34.4 ± 22.7	16/11	15/12	/	
Xie, 2017 [37]	E: 40	54.2 ± 18.9	19/21	42.7 ± 12.0	22/18	/	/	BBS
	C: 40	60.3 ± 12.9	15/25	51.1 ± 8.3	21/19	/	/	
Yang, 2021 [41]	E: 30	60.5 ± 12.8	21/9	75.9 ± 46.0	/	12/18	/	BBS, FAC, fall risk assessed by Tetrax
	C: 30	61.9 ± 13.3	18/12	80.7 ± 40.6	/	10/20	/	
Yao, 2021 [38]	E: 24	67.4 ± 5.8	16/8	20.3 ± 2.6	/	/	/	BBS, COP movement distance and movement area with EC/EO
	C: 20	66.0 ± 5.2	13/7	19.7 ± 3.1	/	/	/	
Zhao, 2022 [39]	E: 20	60.4 ± 12.3	15/5	118.5 ± 69.0	6/14	6/14	Basal ganglia/cerebral hemisphere: 13/7	BBS, TUG, gait parameters (ST-AS, ST-US, SW-AS, SW-US, ST-ASI, SW-ASI, APCOPV-US, APCOPV-AS, EEA-EO, EEA-EC, PPF-EO and PPF-EC) assessed by the ODONATE gait analysis system
	C: 20	54.5 ± 13.9	15/5	100.5 ± 56.1	8/12	9/11	Basal ganglia/cerebral hemisphere: 12/8	

Values are presented as mean ± standard deviation or numbers

*n* number, *M/F* male/female, *H/I* haemorrhagic/ischemic, *L/R* left/Right, *C* control group, *E* experiment group, *BBS* Berg balance scale, *TUG* timed up-and-go test, *PASS* postural assessment scale for stroke patients, *SL* step length, *AS* affected side, *US* unaffected side, *ABC* activities-specific balance confidence scale, *FGA* functional gait assessment, *10MWT* 10-m walking test, *DGI* dynamic gait index, *ST* stance phase, *SW* swing phase, *FAC* functional ambulation category scale, *COP* centre of pressure, *EC* eyes closed, *EO* eyes open, *FM-B* Fugl–Meyer balance scale, *ASI* absolute symmetric index, *APCOPV* anterior–posterior centre of pressure displacement velocity, *EEA* envelope ellipse area, *PPF* proportion of the plantar pressure, *APDCOP* anterior–posterior displacement of centre of pressure, *BBA* Brunel balance assessment

Table 2 provides the intervention details of each trial. Since various intervention protocols were implemented, these trials were classified according to the type of VRT, including GSE or eye–head movements [27–29, 39–41] (40.0%), vestibular sensory stimulation conducted by head movements [33, 35, 36] (20.0%), specific balance exercises combined with eye–head movements [30–32] (20.0%), GSE combined with swivel chair training [37, 38] (13.3%) and swivel chair training [34] (6.67%). The intervention duration of VRT ranged from 1 to 12 weeks, with an average of 4.4 (2.4) weeks. Less than 4-week VRT was used in 4 (26.7%) RCTs [28, 32, 36, 40], 4-week VRT was used in 9 (60.0%) RCTs [27, 29, 31, 33, 34, 37–39, 41] and more than 4-week VRT was used in 2 (13.3%) RCTs [30, 35].

**Table 2:** Intervention detail

**Study (author, year)**	**Experiment group**	**Control group**
Correia, 2021 [28]	Domiciliary intervention based on oculomotor and GSE, and stroke rehabilitation program. Oculomotor and GSE (1) eyes movement between two stationary targets while keeping the head still, (2) tracking exercises while keeping the head still, (3) VOR 1 and (4) VOR 2. Twice a day for 3 weeks.	Stroke rehabilitation program was provided for 3 weeks, according to the identified problems and was based on the clinical reasoning, supported on the neurophysiology, motor control, biomechanics and motor learning theories.
Dai, 2013 [29]	Cawthorne–Cooksey exercises and conventional rehabilitation. The Cawthorne–Cooksey exercises involved side-to-side head turns, up-and-down head movements and gaze movements. Approximately 30 min, 5 days a week for 4 weeks.	Conventional rehabilitation involved PT (passive exercises, active exercises, resistive exercises and ambulation training) and OT (endurance exercise, balance training and ADL training). 1 h for PT and 1 h for OT, 5 days a week for 4 weeks.
Elhamrawy, 2021 [31]	VRT and traditional gait training. VRT included (1) eye movements, (2) head movements, (3) balancing on mat and swivelling eyes, (4) standing on mat, walking on the spot and rotating the head, (5) weight shifting while keeping the sight fixed, (6) standing on foam with eyes closed and head rotation, (7) forward and backward walking with head turning, (8) sitting on a ball with feet on foam, eyes closed and bouncing slightly while turning the head, (9) marching in place. 1–4 weeks: 40 min traditional gait training and 20 min VRT, 4 days a week. 5–8 weeks: traditional gait training, 60 min a day, 4 days a week.	Traditional gait training included strengthening exercises, walking over obstacles, up and down slopes, and 15–20 min of treadmill walking at a speed of 1.2–2.6 km/h. 60 min per day, 4 days a week for 8 weeks.
Guo, 2022 [40]	Vestibular sensory integration training and UR. Vestibular sensory integration training included exercises in various position with head and eyes movements, progressed by eyes open/closed and firm/foam surfaces. 15 min a day, 5 days a week for 3 weeks.	UR include electrical stimulation, PT, OT, hyperbaric oxygen therapy, transcranial magnetic stimulation and specific balance training. Balance training was applied by using a balance instrument (E-LINK, Beijing, China). 30 min a day, 5 days a week for 3 weeks.
Hansson, 2020 [30]	VRT and UR. VRT consisted of (1) standing on a padded mat, feet as close to each other as possible, (2) sitting on a Swiss ball, (3) standing on a trampoline, (4) sitting on a chair or on the edge of a bed. All exercises progressed with eye movements, or/(and) with eyes open/closed, or/(and) with head movements. Twice a week for 3 months.	UR comprised of individually adapted exercises, based on the assessment by the PT. Twice a week for 3 months.
Huang, 2019 [33]	VRT and UR. VRT included head lateral flexion and shift in the coronal plane in sitting and standing. VRT progressed from eyes open/supported to eyes closed/unsupported. 20 min a day, 5 days a week for 4 weeks.	UR based on Bobath and motor learning, included rolling, sitting balance, sit to standing, standing balance training and weight shifting. 50 min a day, 5 days a week for 4 weeks.
Jiang, 2012 [34]	Swivel chair vestibular rotational training and UR. Once a day for 4 weeks.	UR included bed mobility, sitting and standing balance training, gait training and ADL training. 30–60 min, once a day for 4 weeks.
Li, 2022 [35]	VRT and UR. VRT included head lateral flexion and shift in the coronal plane in sitting and standing. VRT progressed from eyes open/supported to eyes closed/unsupported. 20 min a day, once a day for 2 months.	UR included bed mobility, sitting and standing balance training, gait training and ADL training. 30 min a day, once a day for 2 months.
Mitsutake, 2017 [32]	VRT and conventional rehabilitative intervention. VRT consisted of (1) GSE (VOR 1 and VOR 2), and (2) balance exercises: maintain balance while rotating neck and trunk to the right and left, and weight shifting. VRT was progressed from a firm surface to a foam surface, and from eye-open to eye closed conditions. 1–3 weeks: 40 min conventional intervention and 20 min VRT 4–6 weeks: 60 min conventional intervention	Conventional rehabilitative intervention included a range of motion exercise, strengthening exercise, walking indoors and outdoors, and up and down stairs training. 60 min, 7 days a week for 6 weeks.
Wang YM, 2022 [36]	VRT and UR. VRT included head flexion, extension, lateral flexion, rotation (right and left and clockwise/counterclockwise). 7 day a week for 1 week.	UR included neuromuscular electrical stimulation, balance training, gait training, up and down stairs training, aerobic exercise, and lower limbs strengthening exercise. 90 min a day, once a day for 1 week.
Wang YQ, 2022 [27]	VRT and UR. VRT included GSE, eye movement and GSE during transfer. 30 min a day, 5 days a week for 4 weeks.	UR involved PT based on neurodevelopmental theory and OT (upper limb and hand training, activity of daily living training). 5 days a week for 4 weeks.
Xie, 2017 [37]	VRT and UR. VRT consisted of swivel chair rotational training and VOR training. Twice per day, 6 days a week for 4 weeks.	UR included limb exercises and balance training with visual feedback. 6 days a week for 4 weeks.
Yang, 2021 [41]	Vestibulo-ocular reflex exercises and UR. Vestibulo-ocular reflex exercises in standing consisted of eye movements between two targets while keeping the head still, eye-tracking exercises while keeping the head still, VOR 1 and VOR 2. 10 repetitions for each exercise. 5 days a week for 4 weeks.	UR included a customized UR program and specific balance training. 5 days a week for 4 weeks. Customized UR program was provided according to the identified dysfunction and supported by neuroplasticity theory. Balance training was applied by using a balance instrument (Tetrax, Israel).
Yao, 2021 [38]	VRT and UR. VRT included GSE and swivel chair rotational training. 50 min a day, 5 days a week for 4 weeks.	UR included limb exercises, balance board training, standing and gait training. 30 min a day, 5 days a week for 4 weeks.
Zhao, 2022 [39]	GSE and PT. GSE included saccadic, smooth tracking, VOR 1 and VOR 2 exercises. 30 min a day, 5 days a week for 4 weeks.	PT included standing balance training, weight shifting, walking, strengthening exercise and step-up-and-down training. 30 min a day, 5 days a week for 4 weeks.

*GSE* gaze stability exercises, *VOR* vestibulo-ocular reflex, *PT* physiotherapy, *OT* occupational therapy, *ADL* activity of daily living, *VRT* vestibular rehabilitation therapy, *UR* usual rehabilitation

### Risk of bias

The scores of 5 RCTs [28–32] could be found in the official website of PEDro. There were 4 (7.3%) items of the 5 RCTs [28–32] that were inconsistent between reviewers and the PEDro website, and 6 (5.5%) items of the other 10 RCTs [27, 33–41] were inconsistent after assessment by two reviewers. However, all items were agreed upon after consulting the third reviewer. The PEDro scores are shown in Additional file 3: Table S1. The PEDro scores ranged from 5 to 7, with a mean score of 5.9 (0.7). Eleven studies [27, 29, 30, 33–39, 41] (73.3%) with a score of 6 or 7 were considered as high quality. Four studies [28, 31, 32, 40] (26.7%) with a score of 5 were considered as moderate quality. All studies fulfilled the items of eligibility criteria, random allocation, similar baselines, and point estimates and variability. However, only one study [30] (6.67%) showed concealed allocation, and one study [31] (6.67%) did not have between-group comparisons. Participant and therapist blinding were not found in any study and assessor blinding was implemented only in six studies [29, 30, 32, 36, 39, 40] (40.0%).

### Effect of VRT on improving balance

**Fig. 2:**
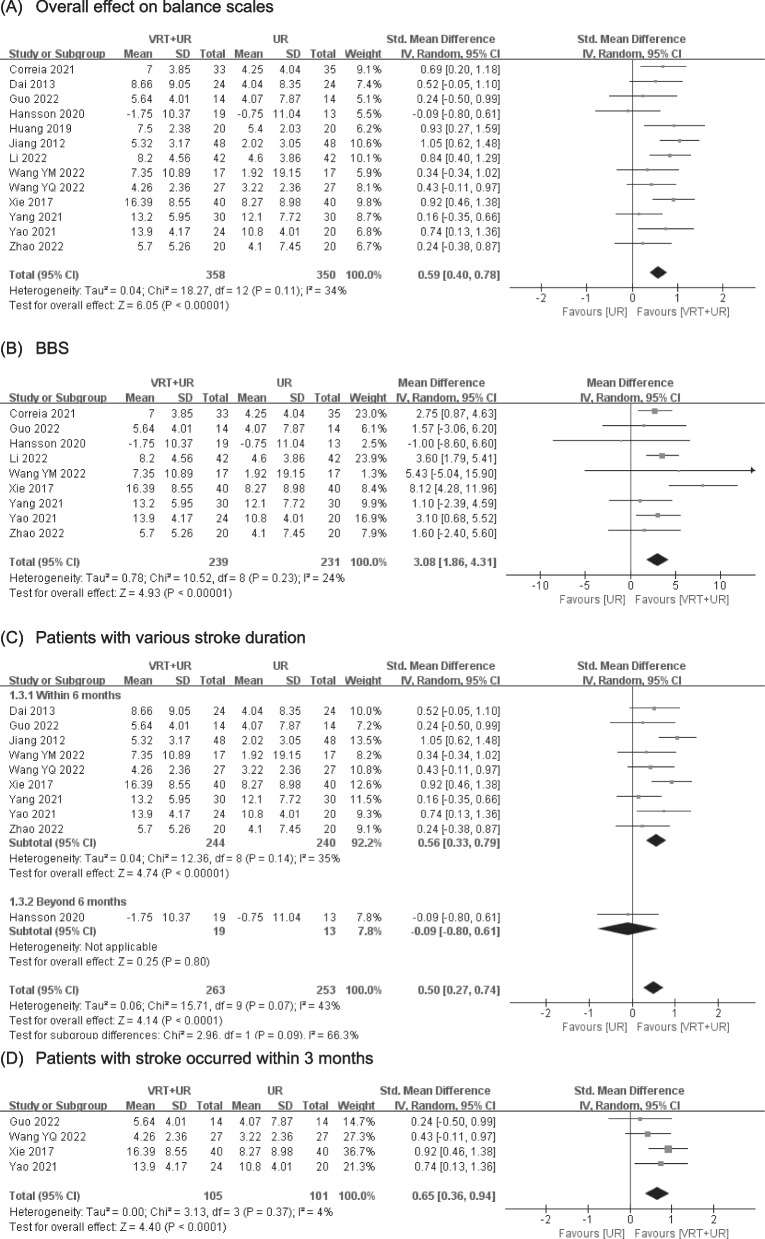
Meta-analysis of VRT on balance (**A**–**D**). VRT vestibular rehabilitation therapy, UR usual rehabilitation, BBS Berg balance scale

Balance was evaluated by using various balance scales and balance equipment. Various balance scales included BBS, FM-B, ABC, BBA and Postural Assessment Scale for Stroke Patients. Thirteen RCTs [27–30, 33–41] with 708 patients were pooled to estimate the overall effect on the balance using balance scales. The pooled SMD showed that VRT significantly improved the balance scores (SMD = 0.59, 95% CI (0.40, 0.78), *p* < 0.00001) with moderate certainty of evidence (Fig. 2A, Table 3). Nine RCTs [28, 30, 35–41] with 470 patients were pooled to estimate the improvements in BBS. The pooled MD indicated a statistically significant improved BBS (MD = 3.08, 95% CI (1.86, 4.31), *p* < 0.00001) with moderate certainty of evidence (Fig. 2B, Table 3). Two RCTs [33, 34] with 136 patients and 2 RCTs [27, 30] with 86 patients were included to estimate changes in FM-B and ABC (Additional file 4: Fig. S1A-B, Table 3). There were significant improvements both on FM-B (MD = 2.74, 95% CI (1.56, 3.91), *p* < 0.00001) and ABC (MD = 7.42, 95% CI (0.83, 14.00), *p* = 0.03) in VRT combined with UR group with moderate certainty of evidence. However, the pooled results of 2 RCTs [38, 39] with 84 patients did not identify a statistically significant decreased the movement area of centre of pressure with eyes open (MD =  −0.70, 95% CI (−2.00, 0.59), *p* = 0.29) or closed (MD =  −3.53, 95% CI (−8.93, 1.88), *p* = 0.20) with low to very low certainty of evidence (Additional file 4: Fig. S1C-D, Table 3). Falls were investigated in 2 studies [28, 29] involving 116 patients. The incidence of falls in the VRT combined with UR group was 0.28 times that in the UR group, but it did not reach the statistically significance (RR = 0.28, 95% CI (0.05, 1.73), *p* = 0.17), with a low certainty of evidence (Additional file 4: Fig. S1E, Table 3).

**Table 3:** Summary of findings of balance and certainty of evidence assessment

**Outcomes**	**Effect (95% CI)**	**No. of participants (studies)**	**Certainty of the evidence (GRADE)**
Overall effect on balance scales	**SMD 0.59** (0.40 to 0.78)	708 (13 studies)	⨁⨁⨁⊝ **Moderate**^a^ due to risk of bias
BBS	**MD 3.08** (1.86 to 4.31)	470 (9 studies)	⨁⨁⨁⊝ **Moderate**^a^ due to risk of bias
FM-B	**MD 2.74** (1.56 to 3.91)	136 (2 studies)	⨁⨁⨁⊝ **Moderate**^a^ due to risk of bias
ABC	**MD 7.42** (0.83 to 14.00)	86 (2 studies)	⨁⨁⨁⊝ **Moderate**^a^ due to risk of bias
COP movement area with eyes open	**MD −0.70** (−2.00 to 0.59)	84 (2 studies)	⨁⊝⊝⊝ **Low**^a,b^ due to risk of bias, imprecision
COP movement area with eyes closed	**MD −3.53** (−8.93 to 1.88)	84 (2 studies)	⨁⊝⊝⊝ **Very low**^a,b,c^ due to risk of bias, inconsistency, imprecision
Number of falls	**RR 0.28** (0.05 to 1.73)	116 (2 studies)	⨁⨁⊝⊝ **Low**^b,d^ due to risk of bias, imprecision
Patients with various stroke duration	**SMD 0.50** (0.27 to 0.74)	516 (10 studies)	⨁⨁⨁⊝ **Moderate**^a^ due to risk of bias
*Within 6 months*	**SMD 0.56** (0.33 to 0.79)	484 (9 studies)	⨁⨁⨁⊝ **Moderate**^a^ due to risk of bias
*Within 3 months*	**SMD 0.65** (0.36 to 0.94)	206 (4 studies)	⨁⨁⨁⊝ **Moderate**^e^ due to risk of bias
Various types of VRT	**SMD 0.59** (0.40 to 0.78)	708 (13 studies)	⨁⨁⨁⊝ **Moderate**^a^ due to risk of bias
*GSE or eye-head movements*	**SMD 0.40** (0.17 to 0.63)	298 (6 studies)	⨁⨁⨁⊝ **Moderate**^a^ due to risk of bias
*Vestibular sensory stimulation conducted by head movements*	**SMD 0.75** (0.43 to 1.07)	158 (3 studies)	⨁⨁⨁⊝ **Moderate**^a^ due to risk of bias
*GSE combined with swivel chair training*	**SMD 0.85** (0.48 to 1.22)	124 (2 studies)	⨁⨁⨁⊝ **Moderate**^a^ due to risk of bias
Various intervention duration of VRT	**SMD 0.59** (0.40 to 0.78)	708 (13 studies)	⨁⨁⨁⊝ **Moderate**^a^ due to risk of bias
*Less than 4 weeks*	**SMD 0.50** (0.15 to 0.85)	130 (3 studies)	⨁⨁⨁⊝ **Moderate**^d^ due to risk of bias
*4 weeks*	**SMD 0.64** (0.40 to 0.89)	462 (8 studies)	⨁⨁⨁⊝ **Moderate**^a^ due to risk of bias
*More than 4 weeks*	**SMD 0.42** (−0.50 to 1.33)	116 (2 studies)	**Very low**^a,b,c^ due to risk of bias, inconsistency, imprecision

GRADE Working Group grades of evidence:

High certainty: Further research is very unlikely to change our confidence in the estimate of effect

Moderate certainty: Further research is likely to have an important impact on our confidence in the estimate of effect and may change the estimate

Low certainty: Further research is very likely to have an important impact on our confidence in the estimate of effect and is likely to change the estimate

Very low certainty: We are very uncertain about the estimate

*CI* confidence interval, *MD* mean difference, *SMD* standardized mean difference, *RR* risk ratio, *GRADE* grading of recommendations assessment, development and evaluation, *BBS* Berg balance scale, *FM-B* Fugl–Meyer balance scale, *ABC* activities-specific balance confidence scale, *COP* centre of pressure, *VRT* vestibular rehabilitation therapy, *GSE* gaze stability exercises

^a^Lack of allocation concealment

^b^Wide 95% CI of pooled effect

^c^Statistical heterogeneity across studies

^d^Lack of allocation concealment and failure to consider the intention to treat principle

^e^Lack of allocation concealment and adequate follow-up

Nine RCTs [27, 29, 34, 36–41] with 484 patients and 1 RCTs [30] with 32 patients were included in the analysis of the efficacy of VRT combined with UR in patients after stroke occurred within 6 months and beyond 6 months (Fig. 2C, Table 3). The result of subgroup analysis showed that patients with stroke occurred within 6 months achieved significant balance improvement in the VRT combined with UR group (SMD = 0.56, 95% CI (0.33, 0.79), *p* < 0.00001) with moderate certainty of evidence. The effects of VRT for patients with onset of stroke within 3 months were estimated in 4 RCTs [27, 37, 38, 40] with 206 patients. There was a statistically significant improvement on balance in the VRT combined with UR group (SMD = 0.65, 95% CI (0.36, 0.94), *p* < 0.0001) with moderate certainty of evidence (Fig. 2D, Table 3).

**Fig. 3:**
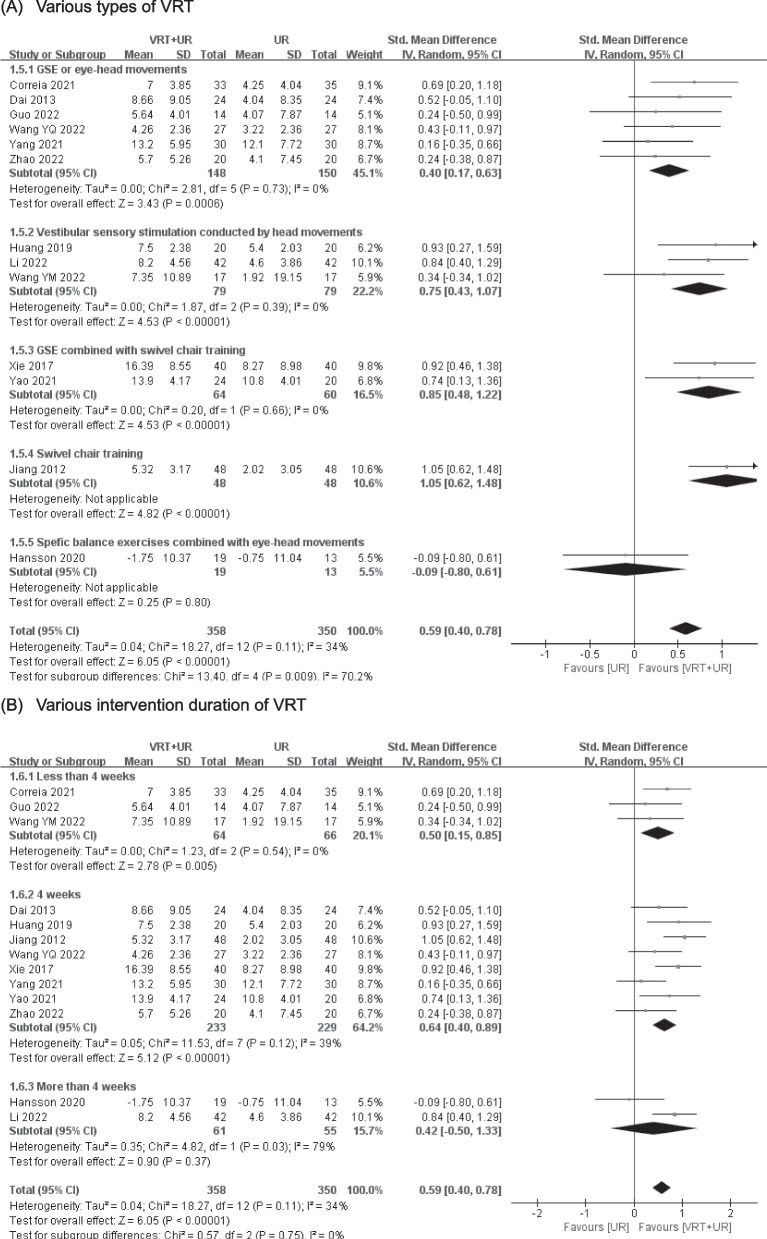
Meta-analysis of various types and intervention duration of VRT on balance (**A**–**B**). VRT vestibular rehabilitation therapy, UR usual rehabilitation, GSE gaze stability exercises

The effects of different types and intervention duration of VRT on balance were estimated in 13 RCTs [27–30, 33–41] with 708 patients. The pooled SMD showed that different types and intervention duration of VRT could significantly improve balance (SMD = 0.59, 95% CI (0.40 to 0.78), *p* < 0.00001) with moderate certainty of evidence (Fig. 3A–B, Table 3). Six RCTs [27–29, 39–41] with 298 patients, 3 RCTs [33, 35, 36] with 158 patients and 2 RCTs [37, 38] with 124 patients were included for subgroup analysis of the effectiveness of GSE or eye-head movements, vestibular sensory stimulation and GSE combined with swivel chair training on balance. There was a statistically significant improvement on balance in the group which VRT were provided by GSE or eye–head movements (SMD = 0.40, 95% CI (0.17, 0.63), *p* = 0.0006), vestibular sensory stimulation conducted by head movement (SMD = 0.75, 95% CI (0.43, 1.07), *p* < 0.00001) and GSE combined with swivel chair training (SMD = 0.85, 95% CI (0.48, 1.22), *p* < 0.00001) with moderate certainty of evidence. Three RCTs [28, 36, 40] with 130 patients, 8 RCTs [27, 29, 33–35, 37, 39, 41] with 462 patients and 2 RCTs [30, 35] with 116 patients were pooled in subgroup analysis to investigate the effect of less than 4-week, 4-week and more than 4-week VRT on balance. The result showed that there was a statistically significant improvement in balance both in less than 4-week VRT group (SMD = 0.50, 95% CI (0.15, 0.85), *p* = 0.005) and 4-week VRT group (SMD = 0.64, 95% CI (0.40, 0.89), *p* < 0.00001) with moderate certainty of evidence. There was not a significant improvement on balance in the more than 4-week VRT group (SMD = 0.42, 95% CI (−0.50, 1.33), *p* = 0.37).

### Effect of VRT on improving gait

**Fig. 4:**
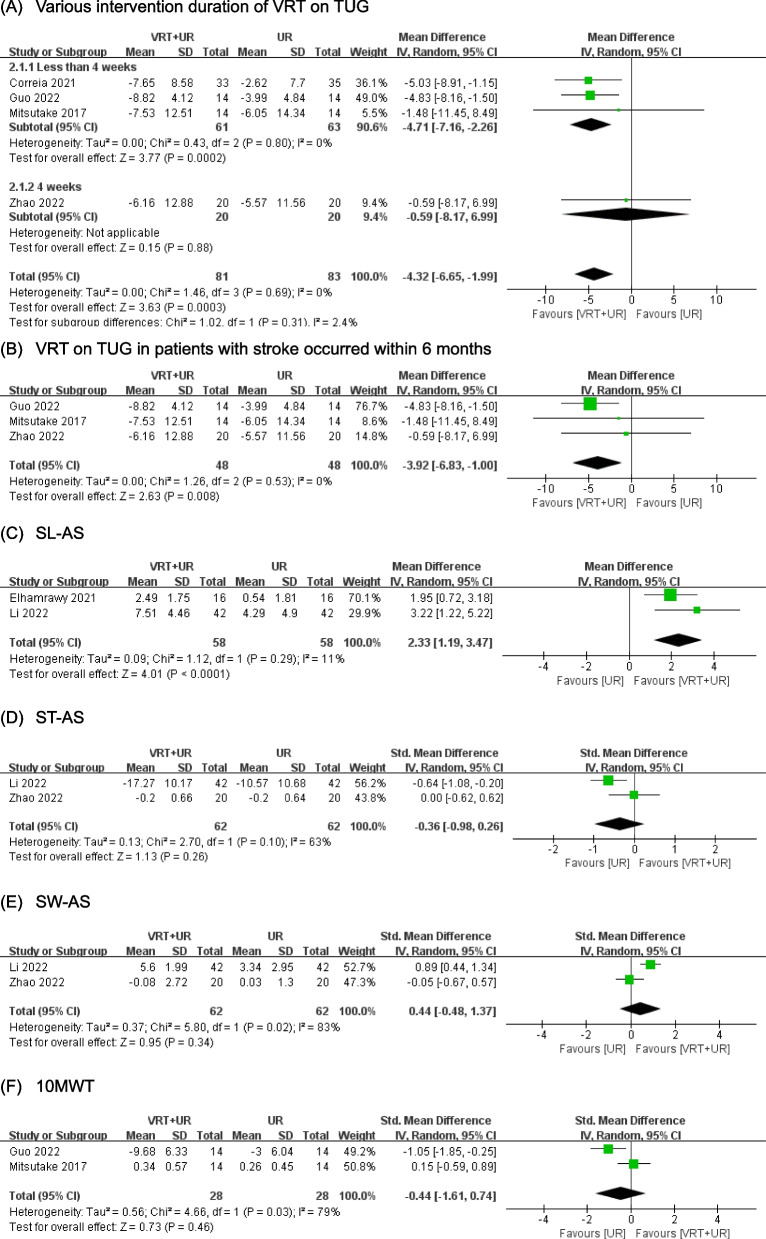
Meta-analysis of VRT on gait (**A**–**F**). VRT vestibular rehabilitation therapy, UR usual rehabilitation, TUG timed up-and-go test, SL step length, ST stance phase, SW swing phase, AS affected side, 10MWT 10-m walking test

Gait was assessed by TUG and gait parameters. Four RCTs [28, 32, 39, 40] with 164 patients were included in examining the improvement in TUG. The pooled MD showed that VRT significantly decreased the TUG (MD =  −4.32, 95% CI (−6.65, −1.99), *p* = 0.0003) with moderate certainty of evidence (Fig. 4A, Table 4). The decreased TUG was observed in the subgroup of less than 4-week VRT (MD =  −4.71, 95% CI (−7.16, −2.26), *p* = 0.0002) with moderate certainty of evidence, instead of the 4-week VRT (MD =  −0.59, 95% CI (−8.17, 6.99), *p* = 0.88). Three RCTs [32, 39, 40] with 96 patients with onset of stroke within 6 months were available for data synthesis. There was a statistically significant improvement on TUG in the VRT combined with UR group (MD =  −3.92, 95% CI (−6.83, −1.00), *p* = 0.008) with moderate certainty of evidence (Fig. 4B, Table 4).

**Table 4:** Summary of findings of gait and certainty of evidence assessment

**Outcomes**	**Effect (95% CI)**	**No. of participants (studies)**	**Certainty of the evidence (GRADE)**
Various intervention duration of VRT on TUG	**MD −4.32** (−6.65 to −1.99)	164 (4 studies)	⨁⨁⨁⊝ **Moderate**^a^ due to risk of bias
* Less than 4 weeks*	**MD −4.71** (−7.16 to −2.26)	124 (3 studies)	⨁⨁⨁⊝ **Moderate**^a^ due to risk of bias
VRT on TUG in patients with stroke occurred within 6 months	**MD −3.92** (−6.83 to −1.00)	96 (3 studies)	⨁⨁⨁⊝ **Moderate**^b^ due to risk of bias
SL-AS	**MD 2.33** (1.19 to 3.47)	116 (2 studies)	⨁⨁⨁⊝ **Moderate**^c^ due to risk of bias
ST-AS	**SMD −0.36** (−0.98 to 0.26)	124 (2 studies)	⨁⊝⊝⊝ **Very low**^d,e,f^ due to risk of bias, inconsistency, imprecision
SW-AS	**SMD 0.44** (−0.48 to 1.37)	124 (2 studies)	⨁⊝⊝⊝ **Very low**^d,e,f^ due to risk of bias, inconsistency, imprecision
10MWT	**SMD −0.44** (−1.61 to 0.74)	56 (2 studies)	⨁⊝⊝⊝ **Very low**^b,d,e^ due to risk of bias, inconsistency, imprecision

GRADE Working Group grades of evidence:

High certainty: Further research is very unlikely to change our confidence in the estimate of effect

Moderate certainty: Further research is likely to have an important impact on our confidence in the estimate of effect and may change the estimate

Low certainty: Further research is very likely to have an important impact on our confidence in the estimate of effect and is likely to change the estimate

Very low certainty: We are very uncertain about the estimate

*CI* confidence interval, *MD* mean difference, *SMD* standardized mean difference, *GRADE* grading of recommendations assessment, development and evaluation, *VRT* vestibular rehabilitation therapy, TUG timed up-and-go test, *SL* step length, *ST* stance phase, *SW* swing phase, *AS* affected side, 10MWT 10-m walking test

^a^Lack of allocation concealment and failure to consider the intention to treat principle

^b^Lack of allocation concealment and adequate follow-up

^c^Lack of allocation concealment and between-groups comparison

^d^Statistical heterogeneity across studies

^e^Wide 95% CI of pooled effect

^f^Lack of allocation concealment

A few studies were included to assess the gait parameters including walking speed, step length of the affected side, stance phase of the affected side and swing phase of the affected side (Fig. 4C–F, Table 4). The pooled MD of 2 RCTs [31, 35] with 116 patients showed that VRT improved step length of the affected side (MD = 2.33, 95% CI (1.19, 3.47), *p* < 0.0001) with moderate certainty of evidence. The pooled results of 2 RCTs [35, 39] with 124 patients did not show a statistically significant improvement in VRT group neither on stance phase (SMD =  −0.36, 95% CI (−0.98, 0.26), *p* = 0.26) nor swing phase (SMD = 0.44, 95% CI (−0.48, 1.37), *p* = 0.34) of affected side. VRT combined with UR could not improve the walking speed measured by 10-m walking test in 2 RCTs [32, 40] with 56 patients (SMD =  −0.44, 95% CI (−1.61, 0.74), *p* = 0.46).

## Discussion

This meta-analytic study included 15 RCTs with 769 patients to investigate the effect of VRT on balance and gait in patients after stroke. The results provided moderate certainty of evidence that VRT could improve the balance and gait in patients after stroke, particularly for patients after stroke that occurred within 6 months.

The VRT improved the overall scores of balance measures, as well as the scores of BBS, FM-B and ABC. There was inadequate evidence for showing the effectiveness of VRT combined with UR in reducing fall incidents of patients after stroke as compared with UR. The point estimate RR was 0.28, indicating a possible protective effect of the VRT combined with UR in reducing the fall incidents, but the 95% CI of RR was 0.05 to 1.73. The most possible underlying reason was the limited sample size in the included studies involving only 116 patients.

The VRT was effective in patients after stroke occurred within 3 months, as well as 6 months, and the pooled statistics of these two categories of patients were very similar. It is known that patients after stroke would achieve the most neurological and functional improvement within the first 3 months, and then the subsequent recovery potential would be limited [42]. However, it is encouraging to observe that patients after stroke could continue to benefit from VRT in balance from 3 month of stroke onset to 6 months.

This study showed that the most effective VRT protocol in improving balance for patients after stroke was GSE combined with swivel chair training, followed by head movement, then GSE or eye–head movements, with 4-week intervention duration. There is strong evidence that VRT prescribed as GSE and/or eye–head movements provide a clear and substantial benefit of gaze and postural stability to individuals with peripheral vestibular dysfunction [7]. Swivel chair vestibular rotational training is based on rotational chair testing, which is considered the most sensitive and reliable technique for quantifying the magnitude of bilateral peripheral vestibular hypofunction [43]. Swivel chair training maximises the physiological stimulation on semi-circular canal by repeatedly changing the flow direction and speed of endolymphatic fluid in each semi-circular canal [44]. This treatment approach may reduce the sensitivity of vestibular system and enhance the tolerance, and promote the compensatory, adaptive and plasticity of the central nervous system [44, 45].

The significant improvement on the TUG scores was observed in the less than 4-week VRT group instead of the 4-week VRT group. Only one trial [39] included in the analysis of 4-week VRT on TUG, showing no significant difference between two groups. More trials with larger sample size may achieve results with statistically significant difference. In the VRT combined with UR group, patients after stroke occurred within 6 months achieved more decreasing TUG scores. For gait parameters, only the positive effects of VRT on the step length of the affected side in patients after stroke were observed. Given the limited data available, subgroup analysis of VRT with different types on gait could not be performed. Due to the heterogeneity of outcome measures, subgroup analysis of the effect of VRT on gait in patients with diverse duration of onset of stroke could not be performed.

### Potential mechanisms of VRT for stroke

The vestibular system is involved in postural control by acting as both a sensory and a motor system, supporting therapeutic applications of VRT in patients after stroke. As a sensory system, the vestibular information closely integrates with somatosensory and visual information to the central nervous system to estimate the position and movement of the entire body as well as the surrounding environment [46, 47]. The vestibular system also contributes directly to motor control. Descending motor pathways such as the vestibulospinal tracts receive vestibular and other types of information to control eye, head and trunk orientation and to coordinate postural movements [46]. Postural adjustments are achieved by reciprocal connections between the vestibular nuclei and the vestibular cerebral cortex, the vestibulocerebellum, reticular formation, spinal cord, superior colliculus and nucleus of cranial nerve XI [46, 48, 49]. The effectiveness of VRT may be achieved by enhancing central processing within the central vestibular nervous system by promoting neural recovery or neuronal plasticity.

### Comparison with other studies

The results of this review are not entirely consistent with previous reviews. A recent systematic review without meta-analysis [17] investigated the effectiveness of VRT on gait performance in patients after stroke. It showed beneficial effects of VRT on gait performance in patients after stroke with very low certainty of evidence. The other systematic review [11] with 12 studies reported the effect of VRT on adult patients with a diagnosis of neurologic disorders. The high clinical and methodological heterogeneity of the included studies precluded meta-analysis in that review. The authors suggested that VRT was safe and could easily be implemented with standard neurorehabilitation in patients with neurologic disorders. However, recommendations on the clinical details of VRT could not be made due to the heterogeneity of treatments and lack of high-quality studies. The differences in the method of analysis and inclusion criteria for participants may explain the somewhat different results of these reviews from those of this present study.

### Implications for clinical practice and research

This meta-analysis provides further support for the effectiveness of VRT in patients after stroke. VRT is recommended in addition to standard stroke rehabilitation to improve the balance and gait in patients after stroke occurred within 6 months. Although a definitive protocol of VRT cannot be recommended for the time being, the most effective VRT in balance improvement appears to be the use of GSE combined with swivel chair training for 4 weeks.

To facilitate future analyses of VRT for patients after stroke, RCTs of higher quality and larger simple size are needed to enhance the certainty of evidence. In addition, stroke characteristics (type, location of lesion, severity and duration of onset) as well as the detailed intervention of VRT prescription should be specified in the future studies.

### Strengths and limitations

The strength of this systematic review included the most comprehensive synthesis of evidence to date on the effects of VRT for patients after stroke. A prespecified protocol registered on PROSPERO was used and the Preferred Reporting Items for Systematic reviews and Meta-Analysis statement was followed. The PEDro scale was used to evaluate the risk of bias, with the GRADE system to appraise the overall certainty of the evidence and present the findings. As there was no language restriction, the language bias would be minimised.

This systematic review had several limitations. The authors could not be reached to obtain the raw data from one potentially eligible trial [50]. The limited use of allocation concealment and assessor blinding in those included trials had introduced bias. The effect of VRT in patients with duration of onset of stroke beyond 6 months were not examined as data were not available for data synthesis in balance and gait measures. Given the limited data available, subgroup analysis of VRT with different types on gait could not be performed. There was also difficulty in categorising the participants into subgroups based on stroke characteristics (type, location of lesion, severity and duration of onset) and the ambulatory status, as these characteristics were not described in detail in most of the included studies.

## Conclusions

There is moderate certainty of evidence supporting the positive effect of VRT on improving balance and gait in patients after stroke, particularly for patients after stroke with onset within 6 months. Higher quality of randomised controlled trials with larger sample size is warranted.

### Supplementary Information

**Additional file 1.** Preferred Reporting Items for Systematic reviews and Meta-Analysis statement checklist.

**Additional file 2.** The search strategy used in PubMed.

**Additional file 3: Table S1.** PEDro Scores of the Included Studies.

**Additional file 4: Figure S1.** Meta-analysis of VRT on other balance outcomes and falls.

## Data Availability

All data generated or analysed during this study are included in this published article and its supplementary materials.

## Abbreviations

ABC: Activities-specific balance confidence scale
BBA: Brunel balance assessment
BBS: Berg balance scale
CI: Confidence interval
FM-B: Fugl–Meyer balance scale
GRADE: Grading of recommendations assessment, development and evaluation
GSE: Gaze stability exercises
MD: Mean differences
PEDro: Physiotherapy evidence database
RCT: Randomised controlled trial
RR: Risk ratio
SD: Standard deviation
SMD: Standardised mean difference
TUG: Timed up-and-go test
UR: Usual rehabilitation
VRT: Vestibular rehabilitation therapy

## References

[1] Harris JE, Eng JJ, Marigold DS, Tokuno CD, Louis CL (2005). Relationship of balance and mobility to fall incidence in people with chronic stroke. Phys Ther.

[2] Mitsutake T, Sakamoto M, Ueta K, Horikawa E (2020). Standing postural stability during galvanic vestibular stimulation is associated with the motor function of the hemiplegic lower extremity post-stroke. Top Stroke Rehabil.

[3] Mitsutake T, Chuda Y, Oka S, Hirata H, Matsuo T, Horikawa E (2014). The control of postural stability during standing is decreased in stroke patients during active head rotation. J Phys Ther Sci.

[4] Lacour M, Helmchen C, Vidal PP (2016). Vestibular compensation: the neuro-otologist’s best friend. J Neurol.

[5] Han BI, Song HS, Kim JS (2011). Vestibular rehabilitation therapy: review of indications, mechanisms, and key exercises. J Clin Neurol.

[6] McDonnell MN, Hillier SL (2015). Vestibular rehabilitation for unilateral peripheral vestibular dysfunction. Cochrane Database Syst Rev.

[7] Hall CD, Herdman SJ, Whitney SL, Anson ER, Carender WJ, Hoppes CW (2022). Vestibular rehabilitation for peripheral vestibular hypofunction: an updated clinical practice guideline from the Academy of Neurologic Physical Therapy of the American Physical Therapy Association. J Neurol Phys Ther.

[8] Martins ECSD, Bastos VH, de Oliveira Sanchez M, Nunes MK, Orsini M (2016). Effects of vestibular rehabilitation in the elderly: a systematic review. Aging Clin Exp Res.

[9] Ricci NA, Aratani MC, Doná F, Macedo C, Caovilla HH, Ganança FF (2010). A systematic review about the effects of the vestibular rehabilitation in middle-age and older adults. Rev Bras Fisioter.

[10] Kundakci B, Sultana A, Taylor AJ, Alshehri MA (2018). The effectiveness of exercise-based vestibular rehabilitation in adult patients with chronic dizziness: a systematic review. F1000Res.

[11] Tramontano M, Russo V, Spitoni GF, Ciancarelli I, Paolucci S, Manzari L (2021). Efficacy of vestibular rehabilitation in patients with neurologic disorders: a systematic review. Arch Phys Med Rehabil.

[12] Acarer A, Karapolat H, Celebisoy N, Ozgen G, Colakoglu Z (2015). Is customized vestibular rehabilitation effective in patients with Parkinson’s?. NeuroRehabilitation.

[13] García-Muñoz C, Cortés-Vega M-D, Heredia-Rizo AM, Martín-Valero R, García-Bernal M-I, Casuso-Holgado MJ (2020). Effectiveness of vestibular training for balance and dizziness rehabilitation in people with multiple sclerosis: a systematic review and meta-analysis. J Clin Med.

[14] Hebert JR, Corboy JR, Manago MM, Schenkman M (2011). Effects of vestibular rehabilitation on multiple sclerosis-related fatigue and upright postural control: a randomized controlled trial. Phys Ther.

[15] Murray DA, Meldrum D, Lennon O (2017). Can vestibular rehabilitation exercises help patients with concussion? A systematic review of efficacy, prescription and progression patterns. Br J Sports Med.

[16] Tramontano M, Medici A, Iosa M, Chiariotti A, Fusillo G, Manzari L (2017). The effect of vestibular stimulation on motor functions of children with cerebral palsy. Mot Control.

[17] Mitsutake T, Imura T, Tanaka R (2020). The effects of vestibular rehabilitation on gait performance in patients with stroke: a systematic review of randomized controlled trials. J Stroke Cerebrovasc Dis.

[18] Liberati A, Altman DG, Tetzlaff J, Mulrow C, Gotzsche PC, Ioannidis JP (2009). The PRISMA statement for reporting systematic reviews and meta-analyses of studies that evaluate healthcare interventions: explanation and elaboration. BMJ.

[19] Physiotherapy Evidence Database. https://pedro.org.au. Accessed 4 June 2023.

[20] de Morton NA (2009). The PEDro scale is a valid measure of the methodological quality of clinical trials: a demographic study. Aust J Physiother.

[21] Maher CG, Sherrington C, Herbert RD, Moseley AM, Elkins M (2003). Reliability of the PEDro scale for rating quality of randomized controlled trials. Phys Ther.

[22] Balshem H, Helfand M, Schunemann HJ, Oxman AD, Kunz R, Brozek J (2011). GRADE guidelines: 3. Rating the quality of evidence. J Clin Epidemiol.

[23] Guyatt G, Oxman AD, Akl EA, Kunz R, Vist G, Brozek J (2011). GRADE guidelines: 1. Introduction-GRADE evidence profiles and summary of findings tables. J Clin Epidemiol.

[24] Higgins JP, Li T, Deeks JJ. Choosing effect measures and computing estimates of effect. In: Higgins J, Thomas J, editors. Cochrane handbook for systematic reviews of interventions version 6.3. Cochrane; 2022. www.training.cochrane.org/handbook. Accessed 4 June 2023.

[25] Wan X, Wang W, Liu J, Tong T (2014). Estimating the sample mean and standard deviation from the sample size, median, range and/or interquartile range. BMC Med Res Methodol.

[26] Deeks JJ, Higgins JP, Altman DG, Chandler J, Cumpston M, Li T, et al. Analysing data and undertaking meta-analyses. In: Higgins J, Thomas J, editors. Cochrane handbook for systematic reviews of interventions version 6.3. Cochrane; 2022. www.training.cochrane.org/handbook. Accessed 4 June 2023.

[27] Wang Y, Ma L (2022). Effect of vestibular rehabilitation on balance function and quality of daily life in stroke patients.

[28] Correia A, Pimenta C, Alves M, Virella D (2021). Better balance: a randomised controlled trial of oculomotor and gaze stability exercises to reduce risk of falling after stroke. Clin Rehabil.

[29] Dai CY, Huang YH, Chou LW, Wu SC, Wang RY, Lin LC (2013). Effects of primary caregiver participation in vestibular rehabilitation for unilateral neglect patients with right hemispheric stroke: a randomized controlled trial. Neuropsychiatr Dis Treat.

[30] Ekvall Hansson E, Pessah-Rasmussen H, Bring A, Vahlberg B, Persson L (2020). Vestibular rehabilitation for persons with stroke and concomitant dizziness - a pilot study. Pilot Feasibility Stud.

[31] Elhamrawy MY, Mohamed S, Bahnasy W, Saif MY, Elkholy A, Said M (2021). Effect of vestibular rehabilitation therapy on spatio-temporal gait parameters in elderly patients with post-stroke hemineglect. Adv Rehabil.

[32] Mitsutake T, Sakamoto M, Ueta K, Oka S, Horikawa E (2017). Effects of vestibular rehabilitation on gait performance in poststroke patients: a pilot randomized controlled trial. Int J Rehabil Res.

[33] Huang L, Zhou K, Liang T, Mai W, Wu Y (2019). The effects of vestibular rehabilitation on trunk control and balancing capacity in stroke patients with Pusher syndrome. Article in Chinese. J Guangxi Med University.

[34] Jiang X (2012). The effects of vestibular rotation combined with rehabilitation training on balance for hemiplegic stroke patients. Article in Chinese. J Qilu Nurs.

[35] Li X (2022). Effect of vestibular function training on balance function and three dimensional kinematics of lower extremities in stroke patients. Article in Chinese. Chin Med Innov.

[36] Wang Y, Zhang W, Zhong Y (2022). Effect of peripheral vestibule training on balance function of rehabilitative period of stroke patients. Article in Chinese. J Chin Rehabil.

[37] Xie Z, Cao Q, Deng S, Chen Y (2017). The effect of intensive vestibular rehabilitation training on balance for hemiplegic stroke patients. Article in Chinese. Chin Manip Rehabil Med.

[38] Yao Y, Liu W (2021). The effect of vestibular rehabilitation training on vertigo and balance function in patients with posterior circulation strokelation stroke. Article in Chinese. Chin Manip Rehabil Med.

[39] Zhao R, Lu J, Xiao Y, Liu X, Wang Y, Xu G (2022). Effects of gaze stabilization exercises on gait, plantar pressure, and balance function in post-stroke patients: a randomized controlled trial. Brain Sci.

[40] Guo Q, Jiang Y (2022). Effects of enhanced vestibular sensory integration training on balance in stroke patients.

[41] Yang L (2021). Effects of vestibulo-ocular reflex training combined with Tetrax on balance for patients with balance dysfunction after stroke. Article in Chinese. J Chin Rura Med.

[42] Hendricks HT, van Limbeek J, Geurts AC, Zwarts MJ (2002). Motor recovery after stroke: a systematic review of the literature. Arch Phys Med Rehabil.

[43] Fife TD, Tusa RJ, Furman JM, Zee DS, Frohman E, Baloh RW (2000). Assessment: vestibular testing techniques in adults and children: report of the Therapeutics and Technology Assessment Subcommittee of the American Academy of Neurology. Neurology.

[44] Wang N, Zhou H, Huang H, Geng D, Yang X, Yu C (2018). Efficacy of SRM-IV vestibular function diagnosis and treatment system in treating benign paroxysmal positional vertigo. Iran J Public Health.

[45] Zhao Y, Wang L, Li W, Sun Y (2022). The value of high intensity stimulation training of semicircular canal of SRM-IV vertigo diagnosis and treatment system in the rehabilitation of vestibular neuritis. Article in Chinese. J Clin Otorhinolaryngol Head Neck Surg.

[46] Lundy-Ekman L, Lundy-Ekman L (2013). Vestibular and visual systems. Neuroscience: fundamentals for rehabilitation.

[47] Joshua AM, Pai S, Joshua AM (2022). Vestibular rehabilitation. Physiotherapy for adult neurological conditions.

[48] Lopez C (2016). The vestibular system: balancing more than just the body. Curr Opin Neurol.

[49] zuEulenburg P, Caspers S, Roski C, Eickhoff SB (2012). Meta-analytical definition and functional connectivity of the human vestibular cortex. NeuroImage.

[50] Tramontano M, Bergamini E, Iosa M, Belluscio V, Vannozzi G, Morone G (2018). Vestibular rehabilitation training in patients with subacute stroke: a preliminary randomized controlled trial. NeuroRehabilitation.

