# Supplementary material for: Vestibular rehabilitation therapy on balance and gait in patients after stroke: a systematic review and meta-analysis
Source: BMC Med. 2023 Aug 25;21:322. doi: 10.1186/s12916-023-03029-9 (PMC10464347; doi:10.1186/s12916-023-03029-9)
Supplement: Supplementary file 2 — Additional file 2. The search strategy used in PubMed. [file 12916_2023_3029_MOESM2_ESM.docx]

The search strategy used in PubMed

#1 stroke [MeSH Terms]

#2 vestibul* [Title/Abstract]

# 3 balance

#4 postur*

#5 walk*

# 6 gait

#7 #3 OR #4 OR #5 OR #6

#8 randomized controlled trial [Publication Type]

#9 randomi* [Title/Abstract]

#10 controlled [Title/Abstract]

#11 trial [Title/Abstract]

#12 #8 OR #9 OR #10 OR #11

#13 #1 AND #2 AND #7 AND #12
