# Supplementary material for: Vestibular rehabilitation therapy on balance and gait in patients after stroke: a systematic review and meta-analysis
Source: BMC Med. 2023 Aug 25;21:322. doi: 10.1186/s12916-023-03029-9 (PMC10464347; doi:10.1186/s12916-023-03029-9)
Supplement: Supplementary file 3 — Additional file 3: Table S1. PEDro Scores of the Included Studies. [file 12916_2023_3029_MOESM3_ESM.doc]

**Table S1. PEDro scores of the included studies.**

| **Study** | **Eligibility criteria** | **Random allocation** | **Concealed allocation** | **Baseline comparability** | **Participants blinded** | **Therapists blinded** | **Assessors blinded** | **Adequate follow-up** | **No missing data or intention to treat analysis** | **Between-groups comparisons** | **Point estimates and variability** | **Total score (/10)** |
| --- | --- | --- | --- | --- | --- | --- | --- | --- | --- | --- | --- | --- |
| Correia, 2021 | Yes | 1 | 0 | 1 | 0 | 0 | 0 | 1 | 0 | 1 | 1 | 5 |
| Dai, 2013 | Yes | 1 | 0 | 1 | 0 | 0 | 1 | 1 | 0 | 1 | 1 | 6 |
| Elhamrawy, 2021 | Yes | 1 | 0 | 1 | 0 | 0 | 0 | 1 | 1 | 0 | 1 | 5 |
| Guo, 2022 | Yes | 1 | 0 | 1 | 0 | 0 | 1 | 0 | 0 | 1 | 1 | 5 |
| Hansson, 2020 | Yes | 1 | 1 | 1 | 0 | 0 | 1 | 0 | 1 | 1 | 1 | 7 |
| Huang, 2019 | Yes | 1 | 0 | 1 | 0 | 0 | 0 | 1 | 1 | 1 | 1 | 6 |
| Jiang, 2012 | Yes | 1 | 0 | 1 | 0 | 0 | 0 | 1 | 1 | 1 | 1 | 6 |
| Li, 2022 | Yes | 1 | 0 | 1 | 0 | 0 | 0 | 1 | 1 | 1 | 1 | 6 |
| Mitsutake, 2017 | Yes | 1 | 0 | 1 | 0 | 0 | 1 | 0 | 0 | 1 | 1 | 5 |
| Wang YM, 2022 | Yes | 1 | 0 | 1 | 0 | 0 | 1 | 1 | 1 | 1 | 1 | 7 |
| Wang YQ, 2022 | Yes | 1 | 0 | 1 | 0 | 0 | 0 | 1 | 1 | 1 | 1 | 6 |
| Xie, 2017 | Yes | 1 | 0 | 1 | 0 | 0 | 0 | 1 | 1 | 1 | 1 | 6 |
| Yang, 2021 | Yes | 1 | 0 | 1 | 0 | 0 | 0 | 1 | 1 | 1 | 1 | 6 |
| Yao, 2021 | Yes | 1 | 0 | 1 | 0 | 0 | 0 | 1 | 1 | 1 | 1 | 6 |
| Zhao, 2022 | Yes | 1 | 0 | 1 | 0 | 0 | 1 | 1 | 1 | 1 | 1 | 7 |

1=Yes, 0=No.
