# Supplementary material for: Vestibular rehabilitation therapy on balance and gait in patients after stroke: a systematic review and meta-analysis
Source: BMC Med. 2023 Aug 25;21:322. doi: 10.1186/s12916-023-03029-9 (PMC10464347; doi:10.1186/s12916-023-03029-9)

(A) FM-B

Study or Subgroup	VRT+UR Mean	VRT+UR SD	VRT+UR Total	UR Mean	UR SD	UR Total	Weight	Mean Difference IV, Random, 95% CI
Huang 2019	7.5	2.38	20	5.4	2.03	20	47.0%	2.10 [0.73, 3.47]
Jiang 2012	5.32	3.17	48	2.02	3.05	48	53.0%	3.30 [2.06, 4.54]
**Total (95% CI)**			**68**			**68**	**100.0%**	**2.74 [1.56, 3.91]**

Heterogeneity: Tau² = 0.27; Chi² = 1.61, df = 1 (P = 0.20); I² = 38%
Test for overall effect: Z = 4.57 (P < 0.00001)

Mean Difference IV, Random, 95% CI

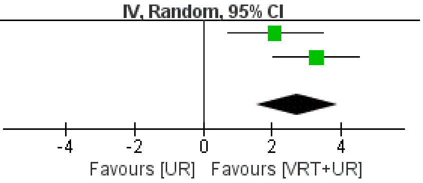

(B) ABC

Study or Subgroup	VRT+UR Mean	VRT+UR SD	VRT+UR Total	UR Mean	UR SD	UR Total	Weight	Mean Difference IV, Random, 95% CI
Hansson 2020	5.92	24.67	19	0	25.13	13	14.0%	5.92 [-11.68, 23.52]
Wang YQ 2022	17.99	14.71	27	10.33	11.75	27	86.0%	7.66 [0.56, 14.76]
**Total (95% CI)**			**46**			**40**	**100.0%**	**7.42 [0.83, 14.00]**

Heterogeneity: Tau² = 0.00; Chi² = 0.03, df = 1 (P = 0.86); I² = 0%
Test for overall effect: Z = 2.21 (P = 0.03)

Mean Difference IV, Random, 95% CI

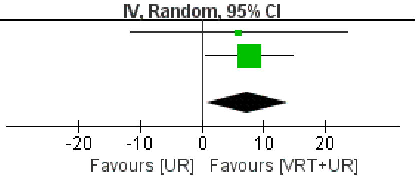

(C) COP movement area with eyes open

Study or Subgroup	VR Mean	VR SD	VR Total	Control Mean	Control SD	Control Total	Weight	Mean Difference IV, Random, 95% CI
Yao 2021	-4.53	3.21	24	-3.39	2.71	20	54.9%	-1.14 [-2.89, 0.61]
Zhao 2022	-0.52	3.72	20	-0.35	2.35	20	45.1%	-0.17 [-2.10, 1.76]
**Total (95% CI)**			**44**			**40**	**100.0%**	**-0.70 [-2.00, 0.59]**

Heterogeneity: Tau² = 0.00; Chi² = 0.53, df = 1 (P = 0.47); I² = 0%
Test for overall effect: Z = 1.06 (P = 0.29)

Mean Difference IV, Random, 95% CI

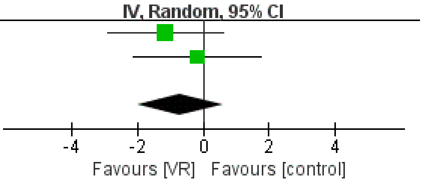

(D) COP movement area with eyes closed

Study or Subgroup	VR Mean	VR SD	VR Total	Control Mean	Control SD	Control Total	Weight	Mean Difference IV, Random, 95% CI
Yao 2021	-12.7	7.47	24	-6	6.87	20	43.1%	-6.70 [-10.94, -2.46]
Zhao 2022	-2.01	1.69	20	-0.88	2.15	20	56.9%	-1.13 [-2.33, 0.07]
**Total (95% CI)**			**44**			**40**	**100.0%**	**-3.53 [-8.93, 1.88]**

Heterogeneity: Tau² = 12.98; Chi² = 6.13, df = 1 (P = 0.01); I² = 84%
Test for overall effect: Z = 1.28 (P = 0.20)

Mean Difference IV, Random, 95% CI

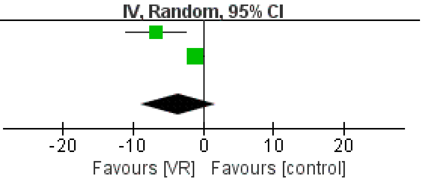

(E) Number of falls

Study or Subgroup	VRT+UR Events	VRT+UR Total	UR Events	UR Total	Weight	Risk Ratio M-H, Random, 95% CI
Correia 2021	0	33	4	35	39.6%	0.12 [0.01, 2.10]
Dai 2013	1	24	2	24	60.4%	0.50 [0.05, 5.15]
**Total (95% CI)**		**57**		**59**	**100.0%**	**0.28 [0.05, 1.73]**
Total events	1		6			

Heterogeneity: Tau² = 0.00; Chi² = 0.62, df = 1 (P = 0.43); I² = 0%
Test for overall effect: Z = 1.37 (P = 0.17)

Risk Ratio M-H, Random, 95% CI

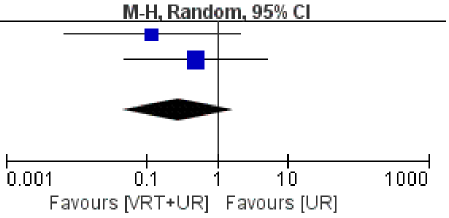

Supplement: Supplementary file 4 — Additional file 4: Figure S1. Meta-analysis of VRT on other balance outcomes and falls. [file 12916_2023_3029_MOESM4_ESM.pdf]
